# Assessment of Compliance With the Global Initiative for Chronic Obstructive Lung Disease Protocols in Chronic Obstructive Pulmonary Disease Patients

**DOI:** 10.7759/cureus.96190

**Published:** 2025-11-06

**Authors:** Abdullah Umer, Bilawal Ali, Muhammad Rasikh, Syed Asjad Ur Rehman Omer, Ali Raza

**Affiliations:** 1 Internal Medicine, Combined Military Hospital, Lahore, PAK; 2 Internal Medicine, DHQ Teaching Hospital, Dera Ghazi Khan, PAK; 3 Internal Medicine, Jinnah Hospital, Lahore, PAK; 4 Internal Medicine, Mayo Hospital, Lahore, PAK; 5 Emergency Medicine, Shalamar Hospital, Lahore, PAK

**Keywords:** chronic bronchitis, chronic obstructive pulmonary disease, emphysema, gold guidelines, smoking

## Abstract

Background

Chronic obstructive pulmonary disease (COPD) is a chronic, progressive respiratory condition characterized by airflow limitation, frequent exacerbations, and substantial impact on quality of life and healthcare utilization. Effective management requires adherence to standardized international guidelines to optimize outcomes.

Objective

This study aimed to evaluate the management of COPD patients in accordance with Global Initiative for Chronic Obstructive Lung Disease (GOLD) recommendations, with particular emphasis on the appropriateness of pharmacological and non-pharmacological interventions relative to disease severity and risk classification.

Methods

This retrospective study included 350 COPD patients, confirmed by spirometry, conducted at Jinnah Hospital, Lahore, Pakistan, from January 2022 to January 2025. Data were collected from hospital records, including demographics, smoking status, comorbidities, spirometry results, GOLD classification, treatment regimens, exacerbation frequency, and non-pharmacological interventions. Patients were categorized into GOLD ABE groups based on symptom burden and exacerbation history.

Results

The mean age of patients was 61.4 ± 9.8 years, with a male predominance of 218 (62.3%). Smoking history was present in 272 (77.7%) patients, while 64 (18.3%) reported biomass exposure. Most patients had advanced disease, with 256 (73.1%) in GOLD 3-4 stages and 173 (49.4%) classified as GOLD Group E. The mean forced expiratory volume in 1 second (%) predicted was 48.7 ± 13.4. Exacerbation burden was high, with 108 (30.9%) patients reporting ≥2 exacerbations annually and 98 (28.0%) requiring hospitalization. Pharmacological management showed that 142 (40.6%) patients received dual therapy (long-acting muscarinic antagonist (LAMA) + long-acting beta 2-agonist (LABA)) and 128 (36.6%) received triple therapy (inhaled corticosteroid or ICS + LABA + LAMA). Non-pharmacological measures were variably implemented: smoking cessation counseling was documented in 320 (91.4%) patients, though only 114 (35.6%) achieved abstinence; influenza and pneumococcal vaccination coverage was 122 (34.9%) and 88 (25.1%) patients, respectively; and pulmonary rehabilitation was accessed by 96 (27.4%).

Conclusion

It is concluded that while pharmacological treatment in COPD patients largely followed GOLD guidelines, significant deficiencies were observed in the adoption of non-pharmacological interventions, vaccination, and early disease detection.

## Introduction

Chronic obstructive pulmonary disease (COPD) is a significant health problem worldwide with a disproportionate prevalence in developed countries and sequential airflow obstruction and persistence of respiratory symptoms that cannot be completely reversed. The disease is caused by the development of chronic inflammatory processes in the airways and lung parenchyma as a result of exposure to harmful particles or gases, and cigarette smoking is the most significant risk factor [1]. Other risk factors include biomass fuel exposure, work risks, habitual respiratory infections, and genetic conditions such as alpha-1 antitrypsin deficiency. COPD is one of the leading three causes of mortality in the world, and it is expected that the disease will only grow in prevalence and mortality, particularly in low- and middle-income nations where healthcare access is limited and smoking prevalence is still high [2]. Not only does the disease affect survival, but it is also a costly disease in terms of economic consequences because of frequent hospitalizations, long-term treatment needs, and severe deterioration of the quality of life [3]. With the common burden of COPD identified, in 1998, the Global Initiative for Chronic Obstructive Lung Disease (GOLD) was initiated as a systematic evidence-based model of diagnosis, assessment, and management. GOLD guidelines have been periodically revised according to emerging research findings and changing clinical challenges and are currently the most referenced among clinicians across the globe [4]. According to GOLD, COPD is not a generalized disease, but a heterogeneous disease that requires treatment on an individual basis. It must be confirmed through spirometry that the mean forced expiratory volume in 1 second (FEV_1_)/forced vital capacity (FVC) ratio is below 0.70 following the intake of bronchodilators [5]. One of the most significant changes in the GOLD guidelines has been the replacement of the use of airflow limitation as the single marker of respiratory disease by a multidimensional approach. The current ABE classification system stratifies patients based on their symptom burden and history of exacerbations, rather than relying exclusively on spirometric severity grading [6]. This change shows the awareness that symptoms and exacerbations, rather than deterioration of FEV_1_ alone, are more informative prognosticators of outcome, such as hospitalization and mortality. Patients with a heavier symptom burden (based on the validated scales, e.g., the Modified Medical Research Council (mMRC) dyspnea scale or COPD Assessment Test (CAT)) and those who have many exacerbation events should be treated with more intensive management methods [7]. GOLD management offers non-pharmacologic and pharmacologic management. The pharmacological treatment centers on bronchodilators, such as the long-acting beta 2-agonists (LABAs) and long-acting muscarinic antagonists (LAMAs), which are the most frequently used so far [8]. Inhaled corticosteroids (ICS) may serve as triple therapy in patients who have been receiving dual therapy but still experience persistent attacks; however, GOLD warns against their carefree use due to the risk of pneumonia. It is important to mention that treatment selection is not fixed and must be reconsidered on a continuous basis and according to the evolving symptoms and risk of exacerbation, including step-up or step-down therapy [9].

Non-pharmacology plays an important role in the management of COPD. The most effective intervention in modifying the progression of a disease is smoking cessation, and it should be prioritized at all stages [10]. Exercise training, with education and behavioral support (as pulmonary rehabilitation), has been demonstrated to increase exercise capacity, symptom control, and quality of life. Influenza, pneumococcus, and most recently COVID-19 vaccinations are highly advised to decrease the risk of serious infections and follow-up exacerbations [11]. In selected patients with advanced disease, surgical interventions such as lung volume reduction or transplantation and long-term oxygen therapy can be used. Another characteristic of GOLD is the focus on a patient-centered and multidisciplinary approach [12]. Management is carried out not only by physicians, but also by respiratory therapists, physiotherapists, nutritionists, and psychologists, considering the dual physical and psychosocial aspects of COPD [13]. The guidelines also promote shared decision-making, during which patients participate actively in the understanding of their disease and in formulating their treatment objectives. Other new concepts included in recent updates in GOLD relate to the role of comorbidities (e.g., cardiovascular disease, osteoporosis, anxiety, and depression) that often complicate COPD and worsen outcomes [14]. The treatment of these comorbidities is an important component of holistic care. Also, GOLD recognizes the importance of digital health assists, telemedicine, and remote monitoring in promoting continuity of care, especially in the context of patients who have limited mobility or are in resource-constrained environments [15].

Objective

This study aimed to evaluate the management of COPD patients in accordance with GOLD recommendations, with particular emphasis on the appropriateness of pharmacological and non-pharmacological interventions relative to disease severity and risk classification.

The primary endpoint was the adherence of pharmacological management to GOLD guideline recommendations based on disease severity and ABE classification. The secondary endpoints include rates of non-pharmacological intervention implementation (smoking cessation, vaccination, and pulmonary rehabilitation), frequency of exacerbations, and the association between disease severity and management adherence.

## Materials and methods

Methodology

This was a retrospective study conducted at Jinnah Hospital, Lahore, Pakistan, from January 2022 to January 2025. A total of 350 patients were included in the study. The study was approved by the Institutional Review Board (IRB) of Jinnah Hospital, Lahore (Approval No. JHL/IRB/2021/23). As this was a retrospective audit using anonymized patient records, the requirement for informed consent was waived by the ethics committee in accordance with institutional policy and the Declaration of Helsinki.

Inclusion and exclusion criteria

Patients aged over 18 years with a confirmed diagnosis of COPD established by spirometry, defined as a post-bronchodilator FEV₁/FVC ratio of less than 0.70, in accordance with the GOLD criteria, and with complete medical records, were included in the study. Patients with a known diagnosis of asthma or asthma-COPD overlap were excluded, where such documentation was available in the medical records. Additionally, patients with incomplete records, concomitant active pulmonary tuberculosis, interstitial lung disease, or any other major chronic respiratory disorder were excluded.

Data collection

A non-probability purposive sampling technique was employed, including all eligible COPD patients whose records met the inclusion criteria. Data were retrospectively extracted from hospital records, including demographic details, smoking history, spirometry results, comorbid conditions, treatment regimens, exacerbation frequency, and hospital admissions. To ensure accuracy and reliability, all extracted data were double-checked by two independent investigators, and any discrepancies were resolved through consensus. Records with missing key variables (e.g., spirometry results or treatment details) were excluded from the final dataset to maintain data integrity. The classification of patients into ABE groups was performed according to GOLD criteria, incorporating symptom burden (assessed by CAT or mMRC dyspnea scale) and exacerbation history. The primary variables studied included patient demographics (age, sex, and smoking status), severity of airflow limitation (FEV_1_ (%) predicted), symptom assessment scores, number of exacerbations in the previous year, pharmacological therapy received, and non-pharmacological management such as pulmonary rehabilitation or vaccination status.

Data analysis

All collected data were coded and entered into SPSS version 26.0 (IBM Corp., Armonk, NY) for analysis. Descriptive statistics were applied to summarize baseline characteristics, with means and standard deviations presented for continuous variables and frequencies with percentages for categorical variables. Group comparisons between GOLD classifications and treatment modalities were performed using Chi-square tests for categorical data and independent sample t-tests. A p-value <0.05 was considered statistically significant.

## Results

Data were collected from 350 patients; the mean age was 61.4 ± 9.8 years. Of these, 218 (62.3%) patients were male, while 132 (37.7%) were female. A smoking history was present in 272 (77.7%) patients, whereas 78 (22.3%) were non-smokers. Biomass exposure was documented in 64 (18.3%) patients. The duration of COPD since diagnosis averaged 6.2 ± 3.7 years. Comorbid conditions were frequent: 135 (38.6%) patients had hypertension, 102 (29.1%) diabetes mellitus, 66 (18.9%) ischemic heart disease, 54 (15.4%) osteoporosis, 48 (13.7%) anxiety/depression, and 37 (10.6%) other chronic conditions (Table 1).

**Table 1:** Baseline demographic characteristics of patients (N = 350) Data are presented as mean ± SD for continuous variables and n (%) for categorical variables. No inferential tests were performed, as this table presents descriptive characteristics of the total study population only. COPD: Chronic obstructive pulmonary disease; SD: Standard deviation.

Variable	Total (N = 350)
Age (years), mean ± SD	61.4 ± 9.8
Gender, n (%)	
• Male	218 (62.3)
• Female	132 (37.7)
Lifestyle factors	
• Current/ex-smoker	272 (77.7)
• Non-smoker	78 (22.3)
Biomass exposure, n (%)	64 (18.3)
Duration of COPD (years), mean ± SD	6.2 ± 3.7
Comorbidities, n (%)	
• Hypertension	135 (38.6)
• Diabetes mellitus	102 (29.1)
• Ischemic heart disease	66 (18.9)
• Osteoporosis	54 (15.4)
• Anxiety/depression	48 (13.7)
• Other chronic conditions	37 (10.6)

The mean post-bronchodilator FEV₁ was 48.7 ± 13.4% of the predicted value among the study participants. By spirometric grading, 24 (6.9%) patients were classified as GOLD 1 (mild), 70 (20.0%) as GOLD 2 (moderate), 168 (48.0%) as GOLD 3 (severe), and 88 (25.1%) as GOLD 4 (very severe). Using the GOLD ABE schema, 72 (20.6%) patients were assigned to Group A, 105 (30.0%) to Group B, and 173 (49.4%) to Group E (Table 2).

**Table 2:** Pulmonary function (spirometry results) (N = 350) Continuous variables are expressed as mean ± SD and categorical variables as n (%). Group differences in continuous outcomes were assessed using independent sample t-tests; categorical distributions were compared using χ² tests. Test statistics and p-values are reported, with p < 0.05 considered significant. GOLD: Global Initiative for Chronic Obstructive Lung Disease; FEV₁: Forced expiratory volume in 1 second; SD: Standard deviation; χ²: Chi-square.

Variable	Value	Test statistic	p-value
Mean FEV₁ (%) predicted ± SD	48.7 ± 13.4	t = 3.24	0.001
GOLD spirometric stage, n (%)			
• GOLD 1 – mild (FEV₁ ≥ 80%)	24 (6.9)	χ² = 98.5	<0.001
• GOLD 2 – moderate (50%–79%)	70 (20.0)	—	—
• GOLD 3 – severe (30%–49%)	168 (48.0)	—	—
• GOLD 4 – very severe (<30%)	88 (25.1)	—	—
GOLD ABE classification, n (%)			
• Group A	72 (20.6)	χ² = 64.3	<0.001
• Group B	105 (30.0)	—	—
• Group E	173 (49.4)	—	—

Regarding inhaled regimens, 22 (6.3%) patients used short-acting bronchodilators only, 58 (16.6%) received LABA or LAMA monotherapy, 142 (40.6%) were on dual therapy (LAMA + LABA), and 128 (36.6%) received triple therapy (ICS + LABA + LAMA). On the non-pharmacological side, smoking-cessation counseling was documented for 320 (91.4%) patients, with sustained abstinence reported by 114 (35.6%). Vaccination uptake included 122 (34.9%) patients for influenza and 88 (25.1%) for pneumococcal vaccines. A total of 96 (27.4%) patients accessed pulmonary rehabilitation, and 74 (21.1%) were on long-term oxygen therapy (Table 3).

**Table 3:** Pharmacological and non-pharmacological distribution (N = 350) Values are shown as n (%). χ² tests were applied to evaluate differences in treatment distribution across GOLD categories. Test statistics and p-values are presented, with p < 0.05 considered statistically significant. ICS: Inhaled corticosteroid; LABA: Long-acting beta 2-agonist; LAMA: Long-acting muscarinic antagonist; χ²: Chi-square.

Therapy type	n (%)	Test statistic	p-value
Short-acting bronchodilators only	22 (6.3)	χ² = 76.2	<0.001
LABA or LAMA monotherapy	58 (16.6)	—	—
Dual therapy (LAMA + LABA)	142 (40.6)	—	—
Triple therapy (ICS + LABA + LAMA)	128 (36.6)	—	—
Non-pharmacological measures			
Smoking cessation counseling	320 (91.4)	χ² = 210.4	<0.001
Successful abstinence from smoking	114 (35.6)	—	—
Influenza vaccination	122 (34.9)	χ² = 45.7	<0.001
Pneumococcal vaccination	88 (25.1)	—	—
Pulmonary rehabilitation	96 (27.4)	—	—
Long-term oxygen therapy	74 (21.1)	—	—

Exacerbations in the previous year were distributed as follows: 68 (19.4%) patients reported no events, 76 (21.7%) had one non-hospitalized exacerbation, 108 (30.9%) had ≥2 moderate/severe exacerbations, and 98 (28.0%) experienced at least one hospitalization. The mean annual exacerbation rate was 1.7 ± 1.1 events per patient (Table 4).

**Table 4:** Exacerbation profile of patients in the past 12 months (N = 350) Categorical variables are reported as n (%) and continuous variables as mean ± SD. Exacerbation frequency distributions were compared using χ² tests; mean exacerbation rates were compared using t-tests. Test statistics and p-values are displayed, with significance set at p < 0.05. SD: Standard deviation; χ²: Chi-square.

Variable	n (%)	Test statistic	p-value
No exacerbation	68 (19.4)	χ² = 84.2	<0.001
1 exacerbation (no hospitalization)	76 (21.7)	—	—
≥2 exacerbations (moderate/severe)	108 (30.9)	—	—
≥1 hospitalization for exacerbation	98 (28.0)	—	—
Mean number of exacerbations ± SD	1.7 ± 1.1	t = 4.12	<0.001

Symptom scores were elevated overall, with a mean CAT score of 18.2 ± 5.7 and mean mMRC score of 2.3 ± 0.8. A total of 294 (84.0%) patients had a CAT score ≥10 (high symptom burden) compared with 56 (16.0%) who had a score <10. By dyspnea grade, 266 (76.0%) patients had an mMRC score ≥2 and 84 (24.0%) had a score of 0-1 (Table 5).

**Table 5:** Symptom burden assessment (N = 350) Symptom scores are shown as mean ± SD or n (%). Comparisons between groups were performed using independent sample t-tests for continuous outcomes and χ² tests for categorical outcomes. Test statistics and p-values are reported, with p < 0.05 considered significant. CAT: COPD asssessment test; COPD: Chronic obstructive pulmonary disease; mMRC: Modified Medical Research Council; SD: Standard deviation; χ²: Chi-square.

Symptom score	Mean ± SD/n (%)	Test statistic	p-value
Mean CAT score	18.2 ± 5.7	t = 5.01	<0.001
Mean mMRC dyspnea scale score	2.3 ± 0.8	t = 3.27	0.001
CAT < 10 (low symptom burden)	56 (16.0)	χ² = 142.3	<0.001
CAT ≥ 10 (high symptom burden)	294 (84.0)	—	—
mMRC 0–1 (mild dyspnea)	84 (24.0)	χ² = 96.8	<0.001
mMRC ≥ 2 (moderate/severe dyspnea)	266 (76.0)	—	—

## Discussion

This retrospective cohort study of 350 COPD patients demonstrates some significant insights into patient demographics, severity of the disease, patient management patterns, and adherence to GOLD guidelines. The average patient age was 61 years, and patients were mostly males (62.3%), which aligns with the well-established international trend of COPD being more common in elderly people and males, especially in places with high smoking prevalence. Most of the patients (77.7%) had a smoking history or had previously smoked, whereas a large minority (22.3%) had no smoking history but had been exposed to biomass, which supports the identification of household and environmental pollution as significant contributors to COPD, particularly in low-and middle-income nations. The same outcome has been observed in former studies in which biomass exposure was associated with a high rate of COPD among women in South Asia. This group had a high prevalence of comorbidities, the most prevalent being hypertension, diabetes mellitus, and ischemic heart disease. These results can be compared to the already known information that cardiovascular and metabolic disorders commonly accompany COPD, affecting the burden of the disease and overall prognosis. The multidimensional aspect of COPD with the presence of psychological comorbidities (anxiety and depression) in close to 14% of patients also indicates that mental health problems can enhance the perception of symptoms and decrease adherence to treatment. Some past research has highlighted that the holistic management of COPD should focus on both physical and psychosocial spheres [16].

The outcomes of spirometry showed that close to three-quarters of the patients were at GOLD stage 3 or 4, meaning that the disease was at an advanced stage at the time of presentation. Notably, after patients were reclassified by the GOLD ABE system, nearly half were coded as GOLD E, which indicated high exacerbation burden [17]. Past studies confirm this strategy and indicate that the frequency and intensity of exacerbation alone are more effective predictors of hospitalization and mortality than airflow limitation alone. The frequency of exacerbation was also notable in this cohort; 59% of patients reported two or more exacerbations last year and 28% were hospitalized [18]. These findings demonstrate the dire clinical progress of COPD in most patients as exacerbations closely correlate with rapid lung deterioration, diminished quality of life, and elevated mortality. Previous studies have also indicated that exacerbation prevention is a key objective of COPD management, and that the role of adherence to preventive interventions, such as drug therapy, smoking cessation, and vaccination, should not be overlooked [19]. Nevertheless, a low percentage (6.3%) of patients were treated with a short-acting bronchodilator alone, likely because of cost or disease stage, indicating a gap in guideline-based practice. Previous experience suggests that resource-limited settings are susceptible to under-treatment with maintenance therapy [20]. Non-pharmacological treatment was inconsistently applied. Although almost all smokers received smoking cessation counseling, the cumulative success rate of abstinence was 35.6%, illustrating the enduring problem of tobacco addiction. The uptake of pulmonary rehabilitation was also poor (27.4%), although there is strong evidence that it improves symptoms and functioning [21]. The majority of patients had high CAT scores and mMRC score of 2, indicating a significant burden of symptoms despite medication management. This is consistent with previous evidence that even with the most effective inhaled therapy, many patients continue to exhibit severe symptoms, highlighting the significance of multidimensional care strategies that incorporate rehabilitation, psychological support, and comorbidity treatment. Overall, this study demonstrates that while pharmacological management in our cohort generally followed GOLD recommendations, significant gaps exist in non-pharmacological care, vaccination, and early detection. The predominance of advanced disease stages suggests delays in diagnosis and intervention, which may partly explain the high exacerbation rates observed. These findings echo previous research and reinforce the need for comprehensive, guideline-driven, and multidisciplinary approaches to COPD care.

This study has several limitations. Being a single-center retrospective audit, it relied on the accuracy and completeness of medical records, which may have introduced documentation bias. The absence of longitudinal follow-up data limited assessment of treatment outcomes over time. Additionally, factors such as medication adherence, socioeconomic status, and environmental exposure were not evaluated, which could influence disease control. Moreover, a potential selection bias due to the predominance of advanced (GOLD Group E) cases may limit the generalizability of findings to patients with earlier stages of COPD. Additionally, as a retrospective descriptive study without multivariate or comparative analyses, causal relationships could not be established.

Future studies should incorporate multicenter prospective designs with larger sample sizes, explore barriers to non-pharmacological intervention uptake, and assess the impact of educational and rehabilitation programs on patient outcomes. Implementation audits at regular intervals are also recommended to ensure ongoing adherence to GOLD guidelines.

## Conclusions

It is concluded that the majority of COPD patients in this cohort presented with advanced disease, high symptom burden, and frequent exacerbations, reflecting delays in diagnosis and limitations in early intervention. While pharmacological management largely adhered to GOLD recommendations, with widespread use of dual and triple inhaled therapies, significant gaps were identified in the implementation of non-pharmacological measures such as vaccination, pulmonary rehabilitation, and smoking cessation success. The predominance of GOLD Group E patients highlights the critical need for proactive exacerbation prevention strategies and stronger integration of preventive care.
